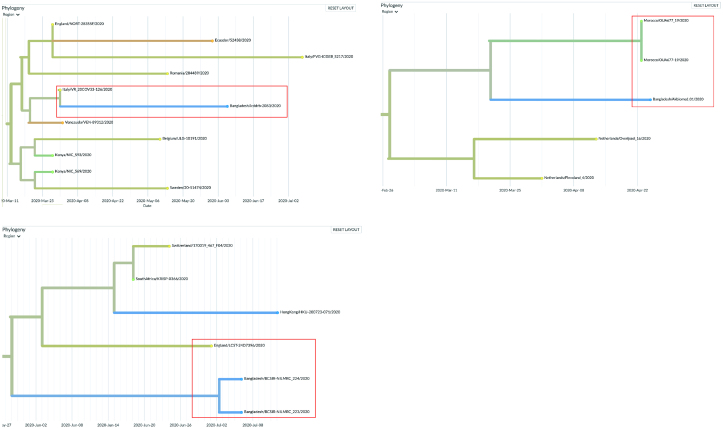# Molecular characterization of SARS-CoV-2 from Bangladesh: implications in genetic diversity, possible origin of the virus, and functional significance of the mutations

**DOI:** 10.1016/j.heliyon.2021.e07866

**Published:** 2021-08-21

**Authors:** Md. Marufur Rahman, Shirmin Bintay Kader, S.M. Shahriar Rizvi

**Affiliations:** a Centre for Medical Biotechnology, Management Information System, Directorate General of Health Services, Mohakhali, Dhaka, 1212, Bangladesh; b International Centre for Diarrhoeal Disease Research, Bangladesh; c Communicable Disease Control, Directorate General of Health Services, Mohakhali, Dhaka, 1212, Bangladesh

**Keywords:** SARS-CoV-2, Mutation, Phylogeny, COVID-19, Bangladesh, D614G, Genome sequence

## Abstract

In a try to understand the pathogenesis, evolution and epidemiology of the SARS-CoV-2 virus, scientists from all over the world are tracking its genomic changes in real-time. Genomic studies can be helpful in understanding the disease dynamics. We have downloaded 324 complete and near complete SARS-CoV-2 genomes submitted in GISAID database from Bangladesh which were isolated between 30 March to 7 September, 2020. We then compared these genomes with Wuhan reference sequence and found 4160 mutation events including 2253 missense single nucleotide variations, 38 deletions and 10 insertions. The C>T nucleotide change was most prevalent (41% of all mutations) possibly due to selective mutation pressure to reduce CpG sites to evade CpG targeted host immune response. The most frequent mutation that occurred in 98% isolates was 3037C>T which is a synonymous change that usually accompanied 3 other mutations that include 241C>T, 14408C>T (P323L in RdRp) and 23403A>G (D614G in spike protein). The P323L was reported to increase mutation rate and D614G is associated with increased viral replication and currently most prevalent variant circulating all over the world. We identified multiple missense mutations in B-cell and T-cell predicted epitope regions and/or PCR target regions (including R203K and G204R that occurred in 86% of the isolates) that may impact immunogenicity and/or RT-PCR based diagnosis. Our analysis revealed 5 large deletion events in ORF7a and ORF8 gene products that may be associated with less severity of the disease and increased viral clearance. Our phylogeny analysis identified most of the isolates belonged to the Nextstrain clade 20B (86%) and GISAID clade GR (88%). Most of our isolates shared common ancestors either directly with European countries or jointly with middle eastern countries as well as Australia and India. Interestingly, the 19B clade (GISAID S clade) was unique to Chittagong, which was originally prevalent in China. This reveals possible multiple introductions of the virus in Bangladesh via different routes. Hence, more genome sequencing and analysis with related clinical data is needed to interpret functional significance and better predict the disease dynamics that may be helpful for policy makers to control the COVID-19 pandemic.

## 1 Introduction

The world is suffering from COVID-19, a devastating pandemic caused by a novel coronavirus originating from Wuhan, China (Zhou et al., 2020). Meanwhile, scientists from all over the world are trying to understand the virus better using various processes including genome sequencing. The first reported complete genome sequence was identified in January 3, 2020 (Tan et al., 2020). More than 141 thousands genomic sequences of SARS-CoV-2 have been submitted in the Global Initiative on Sharing All Influenza Data (GISAID) database (Elbe and Buckland-Merrett, 2017, GISAID-Initiative, 2020).

The genomic sequences revealed that the length of the SARS-CoV-2 viral genome is ~30kb. The longest part of the genome at 5’ end encodes for ORF1ab polyprotein whereas the rest of the genome consists of genes for encoding four structural proteins namely surface (S), envelope (E), membrane (M) and nucleocapsid (N), accessory proteins and other non-structural proteins (NSP) encoded by ORF3a, ORF6, ORF7a, ORF7b, ORF8 and ORF10 genes (Khailany RA and Ozaslan, 2020). The initial assessment of 3 clades indicates distinct geographic distribution. Depending on amino acid changes Förster et al. reported three central variants (A,B,C) of SARS-CoV-2 where A and C being the most common type in Europe and USA and B being the most common type in East Asia (Forster et al., 2020). Pachetti el. al. identified multiple mutation hotspots with geographic location specificity. They identified mutations in RNA dependent RNA polymerase (RdRp) gene which are important as RdRp protein is the target for some proposed antiviral drugs and mutations in the gene may facilitate the virus to escape from those drugs (Pachetti et al., 2020). Yao et al. identified pathogenic variations depending specific SNVs in the Spike glycoprotein (S) changing viral load and cytopathic effects up to 270 folds (Yao et al., 2020). Deletions in the viral genomes are also common phenomena and sometimes are related to severity of the diseases (Armengaud et al., 2020; Holmes, 2009; Pachetti et al., 2020). Still there is a lack of studies to integrate all the deletions in the whole genome of SARS-CoV-2 globally. This may contribute to understand the pathogenic dynamics of the virus over time. The genetic differences among SARS-CoV-2 strains from different locations can be linked with their geographical distributions (Islam et al., 2020).

The clade and lineage nomenclatures for SARS-CoV-2 are changing rapidly. Specific combinations of 9 genetic markers shows 95% of the hCOV-19 data in GISAID can be further classified in major 6 clades named S, L, V, G, GH, GR (Khailany RA and Ozaslan, 2020). Initially the virus was classified into 2, then further into 3 super clades (Forster et al., 2020; Pachetti et al., 2020). A team of scientists has developed an open source bioinformatics and visualization toolkit named Nextstrain (www.nextstrain.org) for real-time tracking of pathogen evaluation including SARS-CoV-2 (nextstrain-github, 2020). Their clade nomenclature is different but supplementary to Rambaut et al., 2020 (GISAID-clade, 2020).

The virus was first reported in Bangladesh on March 8, 2020 as first 3 cases were identified at the Institute of Epidemiology and Disease Research, Dhaka. Currently the country is at the community spread stage and the total number of infected is about 379,738 with 5,555 reported deaths from COVID-19 till 12 October, 2020 (IEDCR, 2020; worldometer, 2020).

It is important to get more sequences from all over the world which will help us for better understanding of the evolution pattern, disease dynamics, phylogeographic distribution of the clades, designing drugs and vaccines. In this paper we tried to determine the phylogenetic relationship of Bangladeshi isolates with other isolates from around the world. This can help us to assume the travelling routes of the virus into Bangladesh as well in other parts of the world. We also tried to know the specific mutational differences in Bangladeshi isolates compared to the reference sequence and whether there is any clinico-pathological significance associated with those mutations.

## 2 Materials and methods

### 2.1 Local sequence retrieval

For retrieval of genome sequences from Bangladesh, we have searched in the GISAID database using search term “Bangladesh” as location. We have downloaded all the relevant sequences from search result in FASTA format and also downloaded patient status metadata, sequencing technology metadata and acknowledgement table separately (accessed on 30 August, 2020).

### 2.2 Mutation analysis

We have used Genome Detective Coronavirus Typing Tool version 1.13 and CoVsurver enabled by GISAID to analyse our query sequences in FASTA format (Cleemput et al., 2020; A∗STAR-Bioinformatics-Institute, 2020). These are freely available online based bioinformatic tools which are validated to identify and reassemble novel Corona virus isolates. Using these tools, we identified both nucleotide and amino acid mutations and similarities compared to SARS-CoV-2 (NCBI Taxonomy ID: 2697049) reference sequence NC_045512 (NCBI) and EPI_ISL_402124 (GISAID).

For functional prediction of mutational changes, we have used two web based tools namely SIFT (Sorting Intolerant From Tolerant) and MutPred2 (Ng and Henikoff, 2003; Pejaver et al., 2017). We also have used USCS SARS-CoV-2 Genome Browser (https://genome.ucsc.edu/cgi-bin/hgGateway?db=wuhCor1) to align mutations along base position and functionally significant areas in the genome (Fernandes et al., 2020). A support vector machine (SVM) based tool namely i-Mutant 3.0 was used for predicting the change of protein stability change and ΔΔ*G* from specific mutations (Capriotti et al., 2005).

### 2.3 Phylogeny analysis

We have used open source bioinformatics visualization platform Nextstrain (nextstrain.org) for phylogeny analysis of our sequences (Hadfield et al., 2018). Pairwise sequence alignment and clade assignment was done using web based Nextstrain tool Nextclade beta version 0.4.9 (Nextclade, 2020). The GISAID clade identification of the sequences was done using GISAID CoVsurver tool. Further detailed information of different clusters was derived from a preformed interactive web-based tree developed from a subsample of global sequences (~5000) by neighbour joining method in Nextstrain web interface (https://nextstrain.org/ncov/global, date accessed on 30 August, 2020).

## 3 Results

From GISAID database we have found 329 submissions from Bangladesh (accessed on 30 August, 2020). Out of the 329 submissions, 324 were complete/near genomes. Among the complete genomes, 102 were isolated from female patients, 220 were from male patients and for 2 isolates the gender was not reported. The age range of collected samples was between 8 days to 95 years and the median age was 38 years. The submissions came from a total of 9 laboratories and different sequencing platforms were used by different laboratories. A quality control (QC) analysis was done where 7 sequences were flagged as “bad” (private mutation cut-off was set at 20) and 6 sequences were reported to have more than 5 ambiguous mutations.

### 3.1 Mutation analysis with functional significance

Variation analysis from 324 genome isolates revealed a total of 4160 mutation events out of which 4112 are Single Nucleotide Variations (SNVs), 38 deletions (in 30 isolates) and 10 insertions (in 10 isolates). Among the SNVs, 2253 were missense mutations in coding regions, 1216 were synonymous mutations and rest were in non-coding part of the genome (Table 1). Among all the SNVs, the most common change was C>T (~41%) and the second most prevalent change was G>A (~16%). There were 5 large deletions (>50 nucleotide) among which three resulted in deletion of a large portion ORF7a gene and another two deleted the ORF8 gene. The highest number of mutation events (including all SNVs and indels) observed in one isolate (EPI_ISL_445217) was 59 and least was zero as one isolate (EPI_ISL_458133) was identical to the reference genome (NC_045512.3) with 99.4% genome coverage. The average number of mutation events was approximately 13 per isolate.

**Table 1:** Distribution of mutation events along different genomic regions of SARS-CoV-2 Bangladeshi isolates.

Genome segment∗		Base Position	Total mutation	Missense Mutation	Synonymous Mutation	Deletion∗∗	Insertion
Coding region	*ORF1ab*	266–21555	1796	1021	775	2	0
	*S*	21563–25384	528	416	112	3	0
	*ORF3a*	25393–26220	135	101	34	7	0
	*E*	26245–26472	4	3	1	0	0
	*M*	26523–27191	65	14	51	0	0
	*ORF6*	27202–27387	8	6	2	1	0
	*ORF7a*	27394–27759	50	9	41	9	0
	*ORF7b*	27756–27887	8	5	3	0	0
	*ORF8*	27894–28259	70	34	36	2	0
	*N*	28274–29533	949	635	314	0	3
	*ORF10*	29558–29674	13	0	13	0	2
Non-coding region	Intergenic	-	12	-	-	2	1
	5′-UTR	1–265	386	-	-	5	1
	3′-UTR	29675–29903	136	-	-	7	3
**Total**			**4160**	**2253**	**1382**	**38**∗∗	**10**

∗ Genes in displayed in italics.

∗∗ Some deletion events continued over multiple regions.

**Figure 1:**
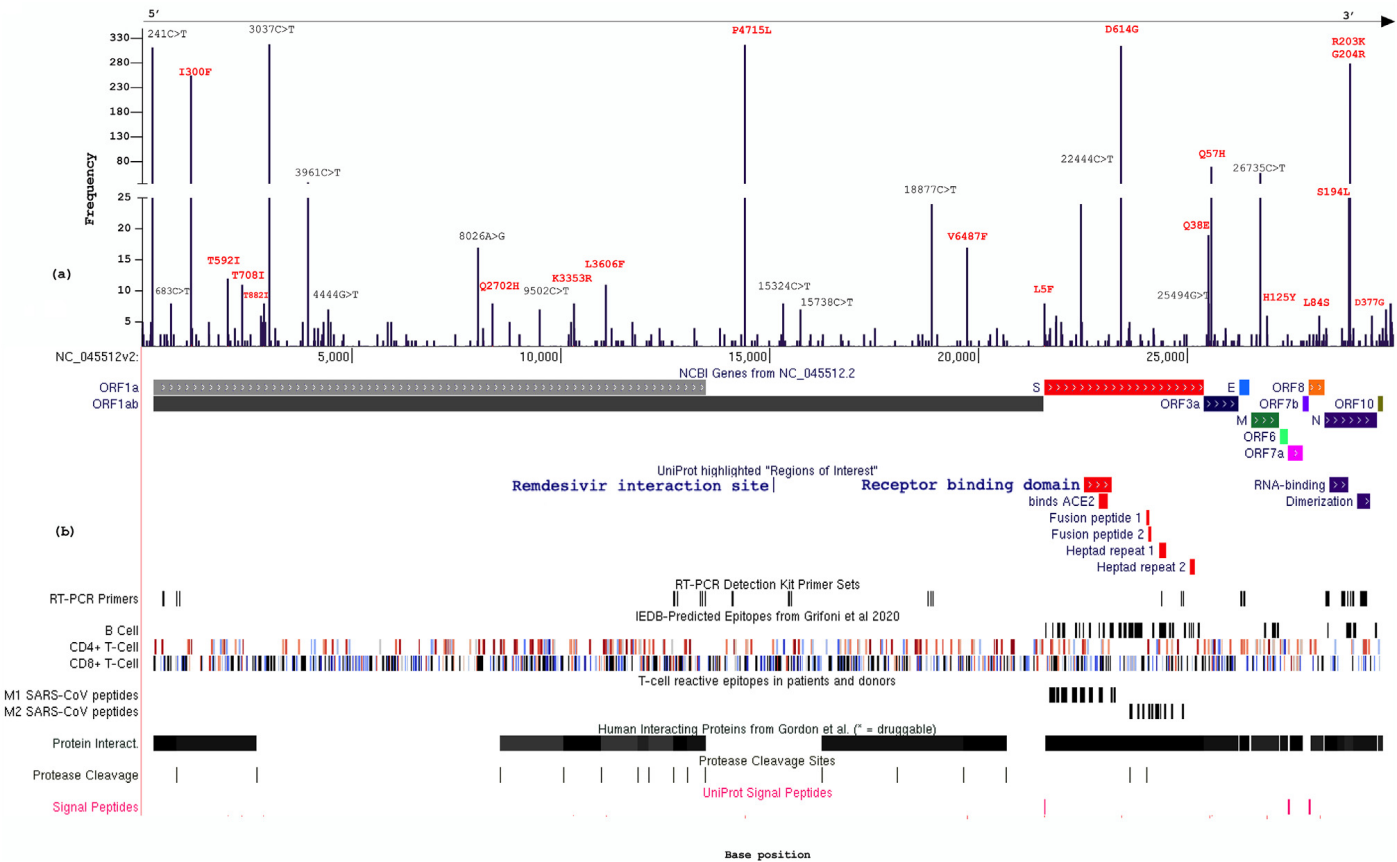
(a) Frequency of SNVs along the base positions of SARS-CoV-2 genome with high frequency (>5) missense SNVs marked red (b) The high frequency missense SNVs aligned (red lines) to different regions of SARS-CoV-2 genome, uniport regions of interest, RT-PCR diagnostic primer set, B-Cell and T-Cell predicted epitope regions, SARS-CoV T Cell epitope regions (M1 and M2 peptides), human protein interaction, protease cleavage and signal peptide regions derived from UCSC genome browser.

The most common mutation in non-coding region was 241C>T, observed in 96% (312) of the isolates. The mutation with highest frequency (~98%) in coding region was 3037C>T (synonymous) and 14408C>T (missense) in ORF1ab gene, and 23403A>G (missense) in S gene. The latter caused D614G amino acid change in the spike protein of the virus. Among the 19 missense SNVs that occurred more than 5 times, 12 were predicted to decrease the stability of their respective protein structure (DDG value less than -0.5 kcal/mol where DDG or Delta Delta G is a measurement for predicting the effect of SNVs on protein stability) and six of the SNVs were predicted to alter protein function. Among these 19 frequent SNVs, 6 were in CD4+ T-Cell epitope predicted regions, 6 were in CD8+ T-Cell epitope predicted regions and 5 were in B-Cell epitope predicted regions. The T592I mutation in ORF1ab polyprotein (NSP2) was strongly predicted for CD8+ T-Cell epitope that was also predicted for altered protein function. The P4715L, one of the highest frequent SNVs, occurred in the RNA dependent RNA polymerase (RdRp) region of ORF1ab polyprotein. Beside these, two of the high frequency (86%) SNVs (R203K and G204R) occurred in COVID-19 diagnostic RT-PCR target and B-Cell predicted epitope regions, of which the later was predicted to cause altered function of the nucleocapsid protein (Figure 1). Further analysis revealed two SNVs (L3606F, H125Y) were predicted to cause altered ordered interface and altered transmembrane protein for ORF1ab polyprotein (NSP6) and Membrane (M) protein respectively where L3606F was predicted to cause gain of sulfation at Y360 position of NSP3 protein (Table 2).

**Table 2:** Variants of SARS-CoV-2 genomes observed in more than 5 isolates.

Genomic change	Type of mutation	Gene	Amino acid change	No. of samples	Structural Prediction Effect (SVM3)	DDG Value (kcal/mol)	Functional prediction effect	Predicted molecular mechanism change	Finding from other studies
241C>T	Non-coding	*5′-UTR*	-	312	-	-	-	-	Frequently observed as co-mutation with 3037C>T (98%), 14408C>T (98%) and 23403A>G (97%) linked with European clade and high infection rate (Mercatelli and Giorgi, 2020; Rahimi et al., 2021)
683C>T	Synonymous	*ORF1ab*	None	8	-	-	-	-	-
1163A>T	Missense	*ORF1ab*	I300F	255	Decrease	-1.79	Tolerated	CD4+ T Cell epitope	Less likely to interact with host factors (Ul Alam et al., 2020)
2040C>T	Missense	*ORF1ab*	T592I	12	Neutral	-0.48	Affect function	CD8+ T Cell epitope (strong prediction)	-
2388C>T	Missense	*ORF1ab*	T708I	11	Neutral	-0.35	Tolerated	CD4+ and CD8+ T Cell epitope	-
2836C>T	Synonymous	*ORF1ab*	None	6	-	-	-	-	-
2910C>T	Missense	*ORF1ab*	T882I	8	Neutral	-0.10	Tolerated	-	-
3037C>T	Synonymous	*ORF1ab*	None	318	-	-	-	-	Frequently observed as co-mutation with 3037C>T (98%), 14408C>T (98%) and 23403A>G (97%) linked with European clade and high infection rate (Rahimi et al., 2021)
3961C>T	Synonymous	*ORF1ab*	None	38	-	-	-	-	Associated with manifestation of diarrhoea and sore throat in patients (Rabbi et al., 2021)
4444G>T	Synonymous	*ORF1ab*	None	7	-	-	-	-	Co-evolving mutation with 8371G>T and 29403A>G (Shishir et al., 2021)
8026A>G	Synonymous	*ORF1ab*	None	17	-	-	-	-	-
8371>T	Missense	*ORF1ab*	Q2702H	8	Decrease	-0.68	Affect function	-	Co-evolving mutation with 8371G>T and 29403A>G (Shishir et al., 2021)
9502C>T	Synonymous	*ORF1ab*	None	7	-	-	-	-	-
10323A>G	Missense	*ORF1ab*	K3353R	8	Neutral	-0.13	Tolerated	CD4+ T Cell epitope	-
11083G>T	Missense	*ORF1ab*	L3606F	11	Decrease	-1.00	Affect function	Altered Ordered interface, Altered Transmembrane protein, Gain of Sulfation at Y3607, CD4+ and CD8+ T Cell epitope	More prevalent in asymptomatic cases (Aiewsakun et al., 2020; Wang et al., 2020)
14408C>T	Missense	*ORF1ab*	P4715L	317	Decrease	-0.83	Tolerated	CD8+ T Cell epitope, RdRp zone,	Frequently observed as co-mutation with 3037C>T (98%), 14408C>T (98%) and 23403A>G (97%) linked with European clade and high infection rate (Rahimi et al., 2021). Associated with higher mutation rate (Pachetti et al., 2020)
15324C>T	Synonymous	*ORF1ab*	None	8	-	-	-	-	-
15738C>T	Synonymous	*ORF1ab*	None	7	-	-	-	-	-
18877C>T	Synonymous	*ORF1ab*	None	24	-	-	-	-	Associated with mutation density in M and E gene (Eskier et al., 2020a)
19723G>T	Missense	*ORF1ab*	V6487F	17	Decrease	-1.55	Tolerated	-	-
21575C>T	Missense	*S*	L5F	8	Decrease	-0.98	Tolerated	-	May increase hydrophobicity of the signal peptide thus facilitate in viral secretion from cell (Zhan et al., 2020) hence increase infectivity (Li et al., 2020). It also increases epitope binding affinity (Guo and Guo, 2020)
21855C>T	Missense	*S*	S98F	6	Neutral	0.00	Tolerated	-	
22444C>T	Synonymous	*S*	None	24	-	-	-	-	Found to co-evolve with 28854C>T and unique to Indian isolates (Banerjee et al., 2020)
23403A>G	Missense	*S*	D614G	315	Decrease	-0.93	Tolerated	B cell epitope	Discussed in detail in the discussion part
25494G>T	Synonymous	*ORF3a*	None	19	-	-	-	-	-
25504C>G	Missense	*ORF3a*	Q38E	8	Decrease	-1.02	Affect function	CD4+ and CD8+ T Cell epitope, transmembrane protein	-
25563G>T	Missense	*ORF3a*	Q57H	30	Decrease	-2.03	Affect function	CD4+ T Cell epitope	Decrease ion permeability of ORF3a channel pore and possibly decrease viral release and immune response (Alam et al., 2020)
26735C>T	Synonymous	*M*	None	29	-	-	-	-	-
26895C>T	Missense	*M*	H125Y	6	Neutral	0.06	Tolerated	CD8+ T Cell epitope, Altered Transmembrane protein Altered Ordered interface	-
28854C>T	Missense	*N*	S194L	25	Neutral	-0.38	Tolerated	B cell epitope	Enhance N-E and decrease N-M interactions thus may promote viral release and attenuate viral assembly (Wu et al., 2021)
28881G>A	Missense	*N*	R203K	279	Decrease	-0.93	Tolerated	B cell epitope, PCR target area, transmembrane protein	Enhance N-E interaction thus may promote viral release and infectivity (Wu et al., 2021).
28882G>A	Missense	*N*	R203K	279	Decrease	-0.93	Tolerated	B cell epitope, PCR target area, transmembrane protein	
28883G >C	Missense	*N*	G204R	279	Decrease	-0.52	Affect function	B cell epitope, PCR target area, transmembrane protein	Enhance N-E interaction thus may promote viral release and infectivity (Wu et al., 2021).
29403A >G	Missense	*N*	D377G	6	Neutral	-0.44	Tolerated	-	-
29742G >A	Non-coding	*3′-UTR*	-	7	-	-	-	-	Create miR-3664-5p binding site but degradation of viral RNA by host protein is unaltered (Mukherjee and Goswami, 2020).
29848T>A	Non-coding	*3′-UTR*	-	8	-	-	-	-	
29850A>T	Non-coding	*3′-UTR*	-	8	-	-	-	-	-
29852A>T	Non-coding	*3′-UTR*	-	7	-	-	-	-	-
29853G>A	Non-coding	*3′-UTR*	-	8	-	-	-	-	-

### 3.2 Mutation analysis of S gene

**Figure 2:**
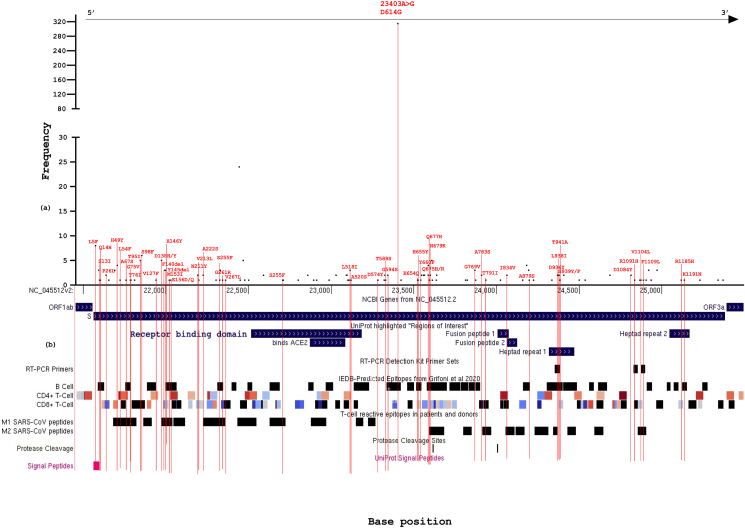
(a) Frequency of SNVs along the base positions of SARS-CoV-2 S gene, missense mutations are annotated in red text (b) The missense SNVs aligned (red lines) to different regions of SARS-CoV-2 S gene, uniport regions of interest, RT-PCR diagnostic primer set, B-Cell and T-Cell predicted epitope regions, SARS-CoV T Cell epitope regions (M1 and M2 peptides), human protein interaction, protease cleavage and signal peptide regions derived from UCSC genome browser.

**Figure 3:**
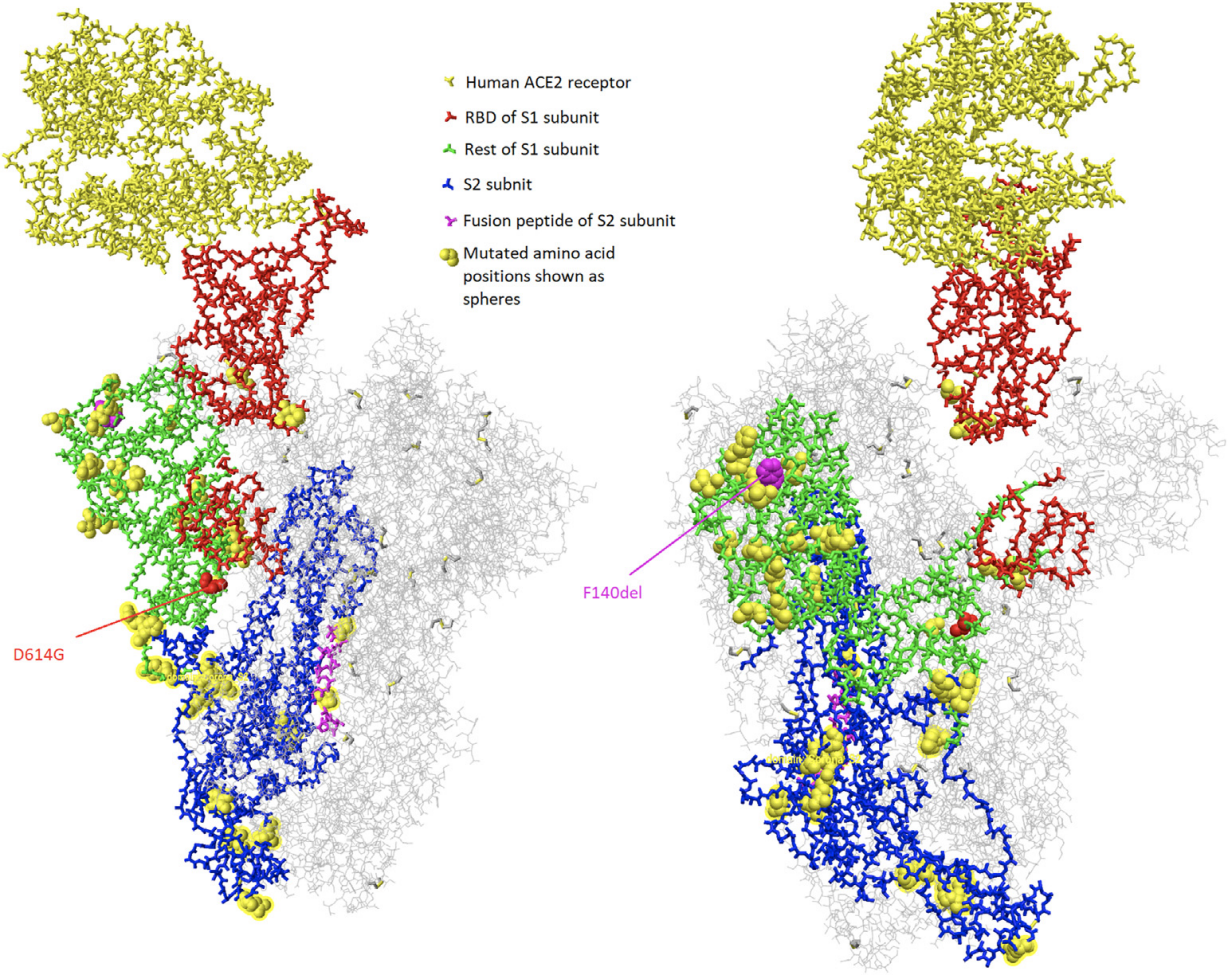
Mutated amino acid positions shown in the human ACE2 receptor bound structure (PDB ID: 6acj) of SARS-CoV-2 spike protein (except L5F, S13I, Q14H, G75V, T76I, Y145del, H146Y, Q675 H/R, Q677H, N679K, I834V, R1185H and K1195N as the regions were not covered in the structure). Mutations are shown in only one chain of the structure.

A separate and detailed analysis of SARS-CoV-2 S gene was done and revealed a total of 530 mutation events among which 414 were missense events and 3 were single amino acid deletions. These mutation events comprised of 56 SNVs and 2 deletions where D614G was the mutation of highest frequency that occurred in 97.2% (315) isolates. Among the SNVs 35 were predicted to decrease protein stability (DDG less than or around -0.5 kcal/mol), 8 were predicted to alter protein function, 15 were in predicted B-Cell epitope region and 28 were in T-Cell predicted epitope region. Individual analysis of these mutations with functional significance is shown in Supplementary Table 1. Three SNVs were found in the receptor binding domain of the spike protein but none of them was in the ACE2 receptor binding part of the protein (Figures 2 and 3).

### 3.3 Phylogeny analysis

**Figure 4:**
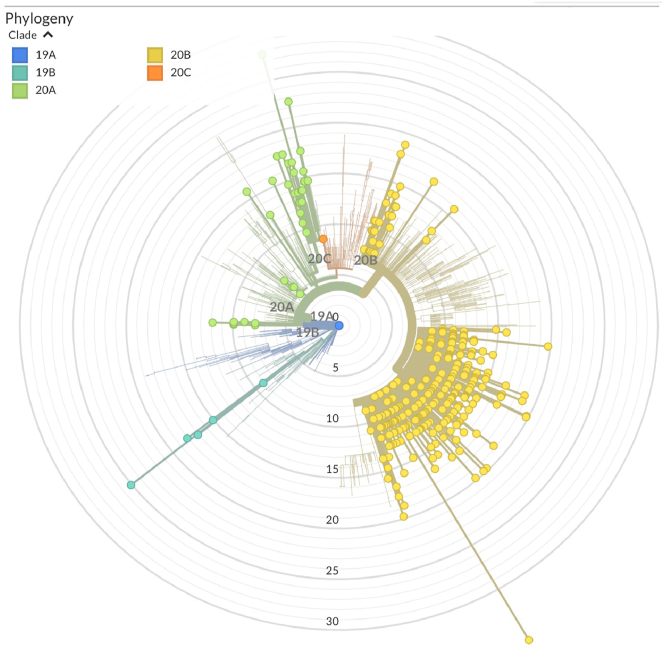
Radial presentation of phylogeny tree formed from 324 SARS-CoV-2 genomes from Bangladesh compared to the reference SARS-CoV-2 sequence shows the isolates belonged to all of the nextstrain clades and the 20B clade is the most prominent.

After phylogeny analysis we have found, our 324 isolates were distributed among all the nextstrain clades where 20B clade was the most frequent (86%) (Figure 4). The 19B clade was unique to Chittagong (5 isolates) and one root clade 19A isolate was reported from Dhaka. There were 24 isolates for which the location data was unknown and only one isolate was found in 20C clade. We also have analysed clade distribution of our sequences according to GISAID nomenclature and found more than 96% of the isolates belonged to the G clade and its two major branches GH and GR clade. The common distinctive feature of these three clades is D614G mutation. About 88% of the sequences clustered in GR clade the distinctive feature of which is G204R mutation in the nucleocapsid protein (Figure 4 and Table 3).

**Table 3:** Location-wise of distribution of isolates from different clades.

Location (Division)	Nextstrain/GISAID Clade	No. of Isolates	Primary Countries
Dhaka	19A/L	1/1	Asia: China/Thailand
	20A/G,GH	6/5,1	N America/Europe/Asia: USA, Belgium, India
	20B/GR,O	77/75,2	Europe: UK, Belgium, Sweden
Chittagong	19B/S	5/5	Asia: China
	20A/G,GH,O	14/2,11,1	N America/Europe/Asia: USA, Belgium, India
	20B/GR	55/55	Europe: UK, Belgium, Sweden
Rajshahi	20A/GH	2/2	N America/Europe/Asia: USA, Belgium, India
	20B/GR	30/30	Europe: UK, Belgium, Sweden
Rangpur	20A/GH	1/1	N America/Europe/Asia: USA, Belgium, India
	20B/GR,G	24/23,1	Europe: UK, Belgium, Sweden
Khulna	20A/O,GH	5/3,2	N America/Europe/Asia: USA, Belgium, India
	20B/GR	22/22	Europe: UK, Belgium, Sweden
Sylhet	20A/GH	2/2	N America/Europe/Asia: USA, Belgium, India
	20B/GR	19/19	Europe: UK, Belgium, Sweden
Barishal	20A/GH	2/2	N America/Europe/Asia: USA, Belgium, India
	20B/G,GR	21/1,20	Europe: UK, Belgium, Sweden
Unknown	20A/G,GH	6/4,2	N America/Europe/Asia: USA, Belgium, India
	20B/GR	16/16	Europe: UK, Belgium, Sweden
	20C/GH	1/1	N America: USA

**Supplementary Figure 1 _spl_1_spl_:**
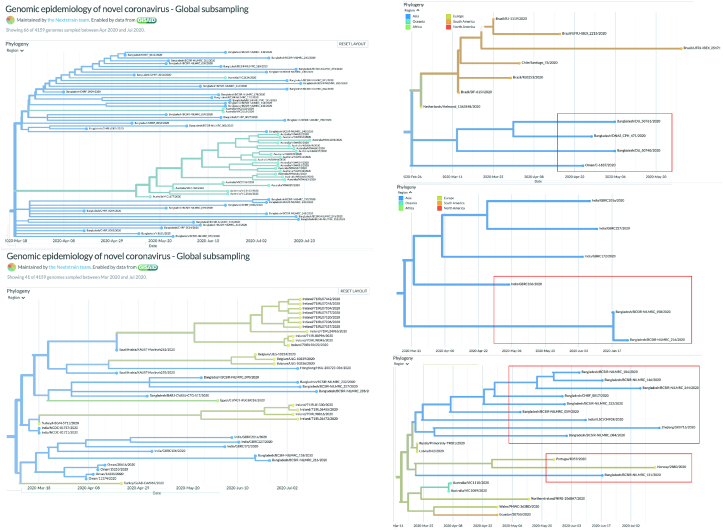


**Supplementary Figure 2:**
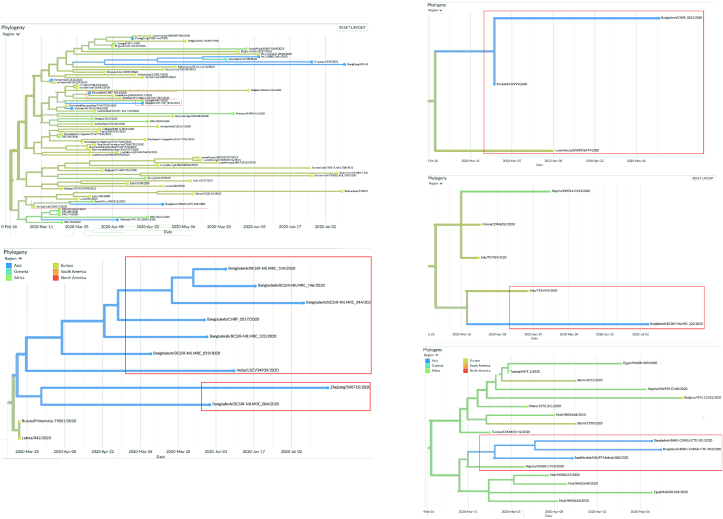


The overall analysis revealed that, most of the isolates shared common ancestors with European countries. A subsampled global phylogeny analysis revealed the largest cluster of Bangladeshi isolates shared common ancestor with some Australian isolates that were reported between mid-May to mid-July. Several other clusters were formed sharing common ancestors with countries including Senegal, Morocco, Egypt, Oman, Saudi Arabia, India, Srilanka, Zhenjiang (China), Portugal, Norway, Luxembourg, Bosnia and Herzegovina, England and Italy (Supplementary Figure 1, Supplementary Figure 2, Supplemantry Figure 3).

## 4 Discussion

**Figure 5:**
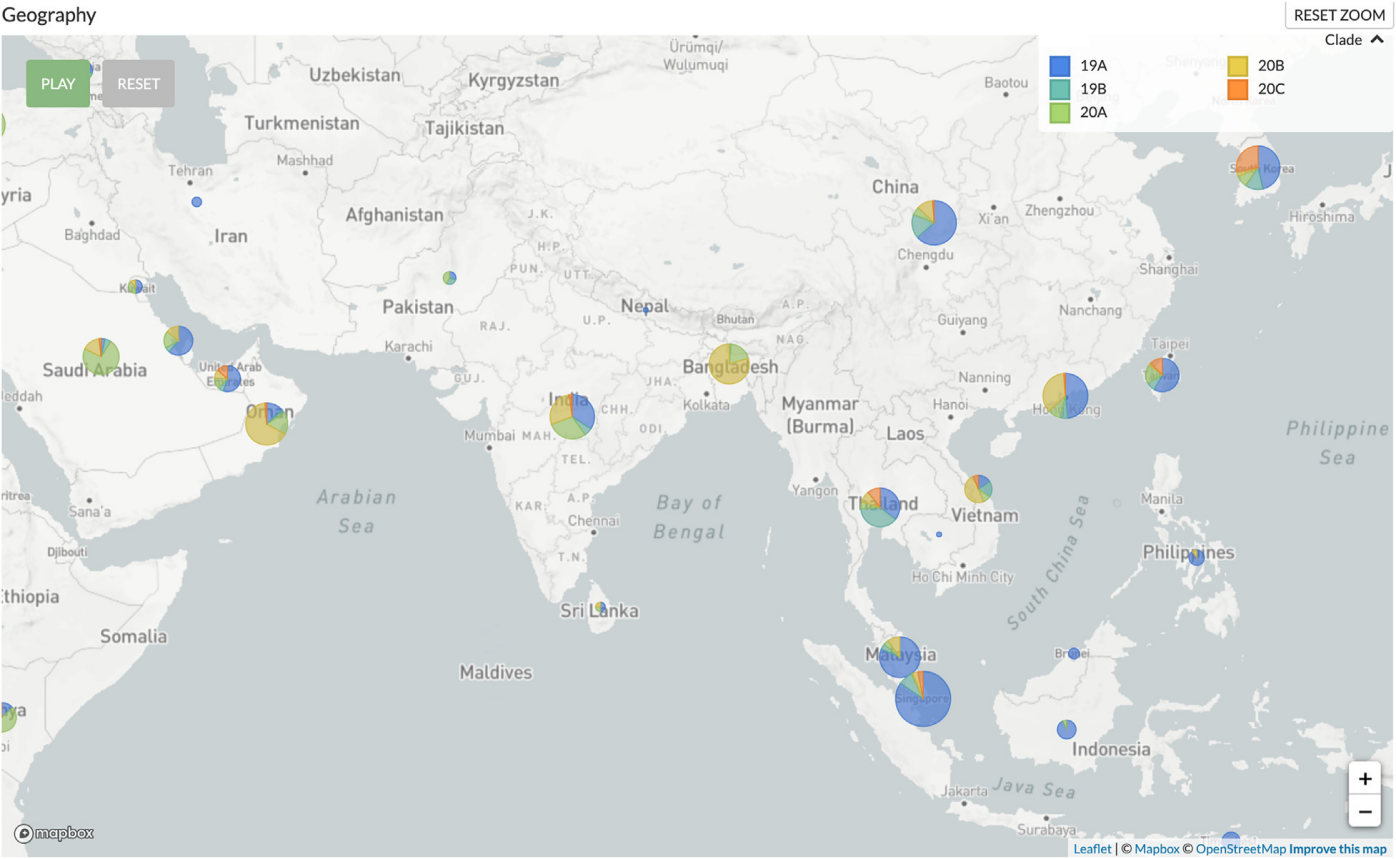
Comparison of clade distribution in different regions of Asia from subsampled global analysis in nextrain.org.

The current SARS-CoV-2 pandemic has changed the world in many ways bringing devastating effects on the society and environment yet we have seen some positive changes and one of which is increased collaboration of scientists and open-source projects from all over the world. The collaborative efforts are making huge impact on research and evidence generation. In our study, we have tried to gather data on genetic evolution and mutational impacts of SARS-CoV-2 those have been isolated and sequenced in Bangladesh. Our analysis revealed multiple introductions of the virus from different regions in our country as the phylogeny tree shows isolates closely related to different countries and regions of the world. Although most of the isolates were related to isolates from Middle Eastern and European countries, this can be explained as a lot of Bangladeshi migrant workers live in those countries including Saudi Arabia, Oman, Belgium and Italy. Many of these migrant workers came back to Bangladesh during first and second quarter of 2020 as number of COVID-19 cases were very high in those regions (Siddiqui et al., 2020; IOM, 2020). Interestingly the largest cluster was formed around Australian isolates but going further back on the tree reveals last common ancestor was also related to the European isolates (Sweden and Switzerland). Hence the abundance of 20B clade is observed in Bangladesh unlike neighbouring India and Pakistan where Asian and North American Clades (19A, 19B, 20B) are more dominant (Figure 5).

In our analysis we have found the most common single nucleotide change was C to T (~41%). This phenomenon was reported earlier and can be explained by selective mutation pressure to reduce CpG sites in the presence of abundant human antiviral proteins including APOBEC3 and ZAP (Wei et al., 2020). The CpG sites are common targets of viral genome and can be recognized by Toll-like receptors (TLRs) that results in release of pro-inflammatory cytokines including type- I interferon, IL-6, IL-12 and TNF-α (Arpaia and Barton, 2011) those play key role in severe COVID-19 including lung tissue damage (Costela-Ruiz et al., 2020). The reducing CpG in SARS-CoV-2 may indicate the mutational change facilitates the viral replication. Similar findings have also been reported in other studies for other RNA viruses relating the CpG motifs and viral replication (Atkinson et al., 2014). Relation of CpG with acute inflammatory response also has been mentioned in case of non-viral gene therapy vectors (Yew and Cheng, 2004). Inverse relation of disease severity with viral load has been reported in a recent study (Argyropoulos et al., 2020). Hence, we think further research is needed focusing the CpG suppression rate in SARS-CoV-2 and its relation with viral load and disease severity which may help designing more potent vaccine and therapeutics.

Large deletion events were observed among some of the isolates that resulted in deletion of most of ORF7a or ORF8 gene products. Three such deletions were between base position 27487 to 27552 (65 nucleotide), 27912 to 28256 (344 nucleotide) and 27472 to 27672 (200 nucleotide). Though these proteins are accessory proteins and not necessary for viral replication, ORF7a was found to interact with human ribosomal transport proteins MDN1 and HEATR3 (Gordon et al., 2020). In SARS-CoV the ORF7a was reported to act as cellular translation inhibitor and apoptosis inducer (Kopecky-Bromberg et al., 2006). Hence deletion of ORF7a may not change viral replication but can alter disease severity by reducing the chance of ORF7a mediated apoptosis. Similar deletions were reported earlier from USA (Addetia et al., 2020). On the other hand, ORF8 protein is least similar to its SARS-CoV homolog and reported to be associated with MHC-I downregulation that facilitates the virus for immune evasion from Cytotoxic T-Cells. This kind of immune evasion facilitates virus to replicate without getting detected by immune cells hence producing less symptoms. Similar mechanism of immune evasion is observed in some chronic infection causing viruses including HIV-1 and Kaposi Sarcoma associated Herpes Virus (KSHV). This may be one of the reasons for the large number of asymptomatic patients, chronic viral shedding even after clinical cure and redetection of virus long after recovery (Zhang et al., 2020b). Recently study found that SARS-CoV-2 ORF8 also can potentially mediate unique immune suppression and evasion mechanisms (Flower et al., 2021).

The 241C>T mutation was one of the most frequent (96%) we have observed in our study. This mutation occurred in 5’ UTR region, so it may not have any functional significance except for reducing CpG sites. Interestingly, this mutation always accompanied 3 other mutations in same isolates. Those mutations include 3037C>T (98%), 14408C>T (98%) and 23403A>G (97%) where the last two are non-synonymous mutations (P4715L in ORF1ab or P323L in RdRp and D614G in Spike protein). This co-occurrence of said mutations are not by chance rather a linkage disequilibrium that has been reported earlier by several studies (Bai et al., 2020; Demir et al., 2020; Mercatelli and Giorgi, 2020). These co-mutations are primary features of GISAID G clade that started rising since February in Europe and now include more than 70% of all SARS-CoV-2 sequences from all over the world (Mercatelli and Giorgi, 2020). The high prevalence of these 4 mutations in Bangladesh also establishes stronger linkage with European isolates. The P323L mutation in RdRp is reported to be associated with increased mutation rate (Begum et al., 2020; Eskier et al., 2020b). Among all 4 mutations the D614G is most studied and reported. Though not in the receptor binding part of Spike protein, multiple studies reported that the D614G mutation provides the SARS-CoV-2 an evolutionary advantage for replication. Studies reported D614G mutation increases infectivity of the virus as it was found to be associated with higher viral load and higher infectious titre (Korber et al., 2020a, 2020b; Zhang et al., 2020a).

The D614 amino acid is located between S1 and S2 junction of spike protein. The cleavage of S1–S2 junction by host protease is crucial for entry into the host cell and multiple cleavage sites enhances the fusion of SARS-CoV with host cell membrane (Belouzard et al., 2009). One study predicted D614G mutation introduces a novel protease (elastase) cleavage site that may enhance the fusion of viral envelop to the host cell membrane hence further facilitate viral RNA entry into the host cell (Bhattacharyya et al., 2020). Studies identified the 614G variant of the virus can get more functional advantage in population with delC variant (rs35074065 site) of TMPRSS2 gene (Bhattacharyya et al., 2020; Russo et al., 2020). This variant is common in Europe, America and South Asia but extremely rare in East Asia according to 1000 Genome project data (NCBI, 2020). This may explain the spread of D614G or G clade in mostly Europe, America and recently in South Asia. While some studies suggested that D614G mutation is associated with higher fatality rate (Becerra-Flores and Cardozo, 2020; Toyoshima et al., 2020), several other studies reported no significant association (Korber et al., 2020b; Cassia Wagner et al., 2020). There is no significant association between viral load and clinical outcome or survival was found in another recent study (Argyropoulos et al., 2020). Considering these analyses it can be said that the current evidence is not clear about the impact of D614G mutation alone on the disease severity and mortality as multiple other stronger factors play role especially age and comorbidity (Grubaugh et al., 2020).

There have been concerns about the impact of D614G mutation on vaccine development but it is clear that the mutation does not take place in the receptor binding region of the spike protein which is the primary target of the neutralizing antibodies. Also, studies suggested that in natural infection, antibodies generated from D614 variant can cross neutralize G614 variant viruses. Hence this is unlikely that the mutation will have a drastic effect on the immunogenicity of the virus and less like to have any impact on vaccine efficacy (Grubaugh et al., 2020; Hu et al., 2020; Ozono et al., 2020).

The second most frequent mutation in our analysis was a tri-nucleotide change resulting in two amino acid changes which are R203K and G204R in N protein. Our analysis revealed these mutations occurred in a PCR target area and B-cell epitope region. Though functionally tolerated, this change was predicted to decrease the protein stability. This finding is similar with other studies those reported R203K and G204R destabilizes N protein structure but may enhance interaction with SARS-CoV-2 Envelop protein that may promote viral release (Rahman et al., 2020; Wu et al., 2021).

The I300F mutation (occurred in 78% isolates) was predicted to reduce the stability of NSP2 protein the function of which is not yet confirmed. One study suggested that the amino acid is positioned within the internal groove of the protein and less likely to interact with host factors (Ul Alam et al., 2020). There two other functionally significant mutations which are Q57H in ORF3a and S194L in N protein. Our analysis suggested the Q57H decreases protein stability with altered protein function may result in loss of a CD4+ T Cell epitope similar to other studies (Kim et al., 2020; Wu et al., 2021). One study mentioned that the Q57H mutation in ORF3a protein may decrease ion permeability by creating a tighter constriction in channel pore and possibly decrease viral release and immune response (Ul Alam et al.). The S194L was predicted to have neutral effect in our study with a reduced DDG value and may attenuate viral assembly as reported in an earlier study (Wu et al., 2021).

We have observed several mutations in some of the RT-PCR target regions. Though it is yet unknown that if mismatch in primer template changes the accuracy and precision of RT-PCR based COVID-19 diagnosis, we recommend avoidance of using primers containing mutation prone regions for better diagnosis. Several other low frequency mutations we found to be associated with higher infectivity and manifestation of specific symptoms which are mentioned in Table 2.

As a limitation of our study, we couldn't derive any clinical information of the patients from whom the samples were collected. The functional significance described in this paper are only computational prediction based and may not always reflect clinical scenario. Also, the genomic sequences were derived using different sequencing platforms (i.e. Illumina, Ion Torrent etc.) and methods (Sanger and Next-generation sequencing) by different laboratories which may have impacted the quality of the sequences hence impacted our analysis. We have found one sequenced that has no mutation compared to the reference sequence which is very unlikely and may possibly be a submission error as the sample was collected long after the original Wuhan outbreak. We hope our findings will create scopes for further research specially including clinical data and also help identifying changes in pathogenicity and infectivity pattern of the virus.

## Declarations

### Author contribution statement

Dr. Md. Marufur Rahman: Conceived and designed the experiments; Performed the experiments; Analyzed and interpreted the data; Contributed reagents, materials, analysis tools or data; Wrote the paper.

Dr. Shirmin Bintay Kader: Performed the experiments; Analyzed and interpreted the data; Contributed reagents, materials, analysis tools or data; Wrote the paper.

Dr. S M Shahriar Rizvi: Conceived and designed the experiments; Analyzed and interpreted the data; Contributed reagents, materials, analysis tools or data; Wrote the paper.

### Funding statement

This work was supported by the Centre for Medical Biotechnology (CMBT), Management Information System, Directorate General of Health Services, Bangladesh.

### Data availability statement

Data included in article/supp. material/referenced in article.

### Competing interest statement

The authors declare no conflict of interest.

### Additional information

No additional information is available for this paper.

## Figures and Tables

**Supplemantry Figure 3:**